# Oculomotor rehearsal in visuospatial working memory

**DOI:** 10.3758/s13414-022-02601-4

**Published:** 2022-11-22

**Authors:** Siobhan M. McAteer, Anthony McGregor, Daniel T. Smith

**Affiliations:** Department of Psychology, Durham University, Durham, UK

## Abstract

The neural and cognitive mechanisms of spatial working memory are tightly coupled with the systems that control eye movements, but the precise nature of this coupling is not well understood. It has been argued that the oculomotor system is selectively involved in rehearsal of spatial but not visual material in visuospatial working memory. However, few studies have directly compared the effect of saccadic interference on visual and spatial memory, and there is little consensus on how the underlying working memory representation is affected by saccadic interference. In this study we aimed to examine how working memory for visual and spatial features is affected by overt and covert attentional interference across two experiments. Participants were shown a memory array, then asked to either maintain fixation or to overtly or covertly shift attention in a detection task during the delay period. Using the continuous report task we directly examined the precision of visual and spatial working memory representations and fit psychophysical functions to investigate the sources of recall error associated with different types of interference. These data were interpreted in terms of embodied theories of attention and memory and provide new insights into the nature of the interactions between cognitive and motor systems.

## Introduction

Visuospatial working memory (VSWM) is the temporary store for the active maintenance of limited amounts of information about objects, with subcomponents for retaining information about their non-spatial features, such as color, and spatial location (Baddeley & Hitch, 1974). Extensive work has shown that there is a tight coupling between VSWM and the oculomotor system during the encoding, maintenance, and retrieval phases (see recent reviews by Heuer et al., 2020; Olivers & Roelfsema, 2020; Van der Stigchel & Hollingworth, 2018). For example, several studies have reported enhanced encoding of visual features for memoranda that were the goal of saccadic eye movements (Bays & Husain, 2008; Hanning et al., 2016), and maintenance and retrieval of object features from VSWM is associated with eye movements towards the spatial location the object previously occupied (van Ede et al., 2019; Williams et al., 2013). The oculomotor system appears to play a similarly important role in the maintenance of spatial information (Pearson et al., 2014; Pearson & Sahraie, 2003; Tremblay et al., 2006). Indeed, performing saccades during the delay period has been shown to selectively impair memory for spatial information, with maintenance of non-spatial information, such as shape and size, being somewhat less affected by oculomotor interference (Ball et al., 2013; Postle et al., 2006). However, the extent to which the role of the oculomotor system in VSWM is specific to the rehearsal of spatial, as opposed to visual (non-spatial), features, and how these underlying representations are affected by saccadic disruption, remains unclear. This study attempts to address these questions by examining how saccadic and attentional disruption affects the precision of VSWM representations.

It has been argued that the oculomotor system aids rehearsal in VSWM through an active control process (Baddeley, 1986). This position has been supported by studies showing that performing eye movements during the retention interval reduces spatial working memory span (Baddeley, 1986; Pearson et al., 2014; Pearson & Sahraie, 2003; Postle et al., 2006). However, other forms of body movement, such as limb movement, also disrupt spatial span (Lawrence et al., 2001; Smyth et al., 1988; Smyth, 1996; Smyth & Scholey, 1994), leading some researchers to argue that rehearsal in VSWM occurs via shifts in spatial attention towards locations held in VSWM (Awh et al., 1998, 1999, 2000). This theory of spatial attention rehearsal argues that, because shifts in spatial attention precede limb and eye movements, performance of these irrelevant behaviors interrupts the shifting of attention towards locations held in VSWM, thereby impairing VSWM maintenance (Awh et al., 1998; Awh & Jonides, 2001; Smyth, 1996; Williams et al., 2013). However, disruption to VSWM due to saccadic interference is greater than disruption caused by limb movement (Lawrence et al., 2004; Pearson & Sahraie, 2003) and by covert orienting (Lawrence et al., 2004; Lilienthal et al., 2018; Pearson & Sahraie, 2003). These findings suggest that oculomotor activity plays a role in VSWM rehearsal that is additional to and independent of covert shifts of attention. As a consequence, the idea that rehearsal takes place via shifts in attention alone has been challenged by several authors, who highlight a specific, functional role of the oculomotor system in the rehearsal of spatial information in VSWM (Belopolsky & Theeuwes, 2009a, b; Postle et al., 2006).

The eye-abduction paradigm has been used to further challenge the argument that VSWM rehearsal takes place via shifts in attention alone. This paradigm involves presenting stimuli to the visual field outside of the oculomotor range, thereby preventing the programming and execution of saccades, without disrupting covert orientation of endogenous attention (Casteau & Smith, 2020; Smith et al., 2012, 2014). Using this paradigm, Ball et al. (2013) showed that disrupting saccade programming selectively decreased spatial span, with no significant effects on working memory for non-spatial features. Subsequently, Pearson et al. (2014) found that interference was greatest when eye abduction was applied during the maintenance interval, considerably reduced when applied during encoding, and absent when during retrieval, consistent with the idea that the oculomotor system has a privileged role in the maintenance of spatial locations in VSWM. These studies, which allow for disentangling attentional mechanisms and oculomotor control processes, support the view that the oculomotor system has a functional role in the rehearsal of spatial information in VSWM, consistent with behavioral (Pearson & Sahraie, 2003; Postle et al., 2006; Tremblay et al., 2006) and neuropsychological evidence (Smith et al., 2021; Smith & Archibald, 2020). However, the reliance on the Corsi blocks task, which emphasizes memory for both spatial location and temporal order rather than the quality of the information maintained in VSWM, means that they could not examine how or why disruption to the oculomotor system affected the representations held in VSWM. As a consequence, the precise nature of interference to spatial and non-spatial components of VSWM representations caused by disruption of the oculomotor system remains unclear.

One way to address the issue of how saccadic disruption affects the underlying representations is to use computational models (e.g., Bays et al., 2009; van den Berg & Ma, 2018; Zhang & Luck, 2008). Peterson et al. (2019) applied Zhang and Luck’s (2008) model to investigate how saccadic and covert attentional interference disrupts VSWM in a location change-detection task. Zhang and Luck’s model (2008) fits a mixture of normal and uniform distributions to response data, giving an estimate of precision and guessing in VSWM. Peterson et al. (2019) found that VSWM was less precise when participants made a saccade to the periphery during the delay period but relatively unimpaired when participants covertly shifted attention to the periphery or maintained central fixation. The effect of saccadic interference was directionally specific: the loss in precision was greatest when changes occurred along the axis of the saccade, with no difference when the change in location occurred along the axis orthogonal to the direction of the saccade. The proposed mechanism for this change in precision was spatial remapping (Bays & Husain, 2007; Wolfe & Whitney, 2015), where the receptive fields of neurons in the visual system are shifted in preparation for a saccade. Within VSWM, this results in the representation of maintained information being remapped in the direction of the saccade (Peterson et al., 2019). This finding suggests that the oculomotor system plays a functional role in rehearsal of spatial information in VSWM by maintaining the precision of the representations of the spatial location of objects.

Although Peterson et al. (2019) have advanced understanding of the role of the oculomotor system in VSWM and discussed potential mechanisms underlying disruption of VSWM due to saccadic disruption, their methods and analyses were somewhat limited in their ability to allow detailed examination of *how* performing delay-period saccades change the VSWM representations. To probe precision, Peterson et al. (2019) varied the magnitude of the spatial change to be detected. However, because responses on this task are binary, the quality of the representation cannot be examined in depth. The degree of noise present within the representations must be inferred from a discrete response space, rather than the distribution of response error, thereby providing limited insight into the sources of recall error. One widely used alternative method is to use a continuous report task, where participants reproduce a feature of a probed stimulus along a continuous dimension, for example reporting the color of the stimulus on a color wheel (Wilken & Ma, 2004). This task permits a more complete insight into the underlying VSWM representation by characterizing response error more thoroughly. Peterson et al. (2019) opted against using a continuous report task, arguing that, because it relies on manual responding, the delay-period interference tasks might have biased subsequent motor planning at response, which may have reduced their ability to isolate the effects of the interference tasks on VSWM. However, the primary function of VSWM is to guide subsequent behavior and aid successful task completion (Baddeley, 2010; Manohar et al., 2017), so biases in motor planning during responding could be argued to reflect changes in the underlying VSWM representation.

Additionally, in Peterson et al.’s (2019) study, participants were required to remember information about only three stimuli. It has reliably been shown that VSWM performance is affected by the number of items retained, with precision in memory of non-spatial and spatial features decreasing monotonically as set size increases (Bays et al., 2009; Schneegans & Bays, 2016). Although saccadic and attentional interference decrease spatial span (Lawrence et al., 2004; Pearson & Sahraie, 2003), it is not clear whether the interference effects depend on how many items must be retained. Previous work has examined how working memory span or memory for a limited number of items is affected by different interference tasks (Lawrence et al., 2004; Pearson et al., 2014; Pearson & Sahraie, 2003; Peterson et al., 2019). However, examination over a range of set sizes allows more specific examination of the interference effects and how these might depend on how many objects are being retained in VSWM.

Finally, the application of Zhang and Luck’s (2008) mixture model is problematic. This model has been criticised for presenting a simplistic overview of sources of error in VSWM because it assumes that all incorrect responses are guesses, occurring as a result of items not being maintained in VSWM (Bays et al., 2009; Ma, 2018). Bays et al.’s (2009) mixture model extends Zhang and Luck’s (2008) model and accounts for the probability of reporting a non-target (misbinding), which can occur due to incorrectly binding non-spatial and spatial features together in VSWM. When the probability of misbinding is included in modelling, the amount of variance accounted for by guessing is significantly decreased (Bays et al., 2009), suggesting that it is an important source of error to consider in VSWM responses (Bays, 2016). From Peterson et al.’s (2019) method, misbinding errors could not be analyzed because memory items were identical so there was no potential for misbinding errors to occur. Nonetheless, it may be premature to conclude that saccadic interference increases the probability of guessing without first examining whether misbinding errors are also affected.

### Aim of current study

The current study aimed to address these criticisms and build on prior work (Pearson et al., 2014; Pearson & Sahraie, 2003; Peterson et al., 2019) to examine how the representations of spatial and non-spatial features in VSWM are affected by delay-period activation of the oculomotor system. We examined the effects of overt and covert shifts of attention on spatial and non-spatial memory representations using a continuous report task. Bays et al.’s (2009) mixture model was used to analyze the results. We carried out two experiments, one probing memory for location and the other memory for color, to more directly examine how VSWM representations change due to saccadic disruption and to investigate the hypothesis that the oculomotor system plays a specific and critical role in rehearsal of spatial information in VSWM.

## Experiment 1

Experiment 1 attempted to examine the effects of delay-period saccadic interference (overt attentional shift) and covert shifts of attention on the representation of spatial information in VSWM. Given that delay-period saccades have been shown to interfere with working memory for spatial locations (e.g., Pearson & Sahraie, 2003; Peterson et al., 2019; Postle et al., 2006), and that shifting covert, endogenous attention is largely independent of the oculomotor system (Casteau & Smith, 2019, 2020; Smith & Schenk, 2012), we proposed the following hypotheses:

1. There will be a main effect of interference task type such that imprecision, misbinding, and guessing will be greatest when the oculomotor system is activated, i.e., in the overt attentional shift, compared to when central fixation is maintained. Although we expect that there will be disruption in the covert attentional shift condition, we expect that disruption in this condition will be smaller than that caused by overt shifts in attention because the oculomotor system is not being activated.
2. There will be a main effect of set size such that imprecision and misbinding will increase with set size, consistent with previous work (Bays et al., 2009; Schneegans & Bays, 2016).
3. The effect of overt attentional shifts on imprecision will be greater in this experiment compared to the effect of overt attentional shifts on imprecision in Experiment 2, which probed memory for color, because the oculomotor system is selectively involved in memory for spatial locations (Ball et al., 2013; Pearson et al., 2014).

We also explored the interaction between set size and interference type, but made no specific predictions regarding this effect.

### Method

#### Participants

An a priori power analysis was carried out using G*Power 3.1 (Faul et al., 2009) to determine how many participants are needed to carry out mixed ANOVA to detect a significant within-between interaction between task and interference task type on precision (Hypothesis 3). Based on unpublished pilot data, this interaction has an effect size of ${\eta}_p^2$ = 0.13 (Cohen’s *f* = 0.39). Based on this, an alpha of .05, and 95% power, the power analysis suggested a total sample size of 24 participants, 12 per experiment. Data collection was finished when 12 usable data sets were obtained.

Undergraduate participants enrolled on Psychology courses at Durham University received participant pool credit for their time. We received ethical approval from Department of Psychology Research Ethics Committee (reference: PSYCH-2019-10-28T15:23:58-lckd86).

#### Design

A within-subjects design was used. The independent variables were interference task type (three levels: overt attentional shift, covert attentional shift, and central fixation) and set size (three levels: one, two, and four items). The dependent variables were imprecision, and the probabilities of reporting the target, misbinding, and guessing.

Participants completed one practice block before beginning each interference task type condition. This practice block comprised 15 trials, five of each set size, to familiarize them with the task. The practice block was identical to the experimental blocks, with the exception that participants were shown their own response as well as the correct response on screen. They then completed 450 trials per interference task type condition, resulting in a total of 1,350 trials for the experiment. Trials in each condition were randomized across 30 blocks. Within each interference interference task type condition, each set size was tested 150 times. The order of completing interference conditions was fully counterbalanced across participants, with the set sizes being randomized within each interference task type condition.

#### Stimuli and apparatus

The task was programmed using Matlab R2019a (The Mathworks, Natick, MA, USA), using the psychophysics toolbox (Kleiner et al., 2007). The stimuli consisted of arrays comprising one, two, or four colored dots (visual angle of each dot = 1^∘^), and a fixation cross positioned at the center of the screen (height of fixation cross in visual angle = 0.76^∘^). The location of each dot was randomly sampled from 24 predefined locations equally spaced along circles with radii of 5°, 7.5°, and 10° of visual angle from central fixation. This ensured that the locations of dots had no overlap. Colors of each dot were randomly chosen from a color wheel, with at least 60^∘^ angular separation on the color wheel between each color. This ensured that each location and color were sufficiently distinct and prevented verbal recoding and learning as much as possible. The visual mask comprised 800 colored dots, with colors randomly chosen from the color wheel, filling the annular space 5°–10° of visual angle around central fixation.

The delay-period interference task was a detection task. Participants were shown chevrons (^/v), with sideways chevrons (>/<) inserted into the sequence at a fixed interval after every four up- and down-facing chevrons. These were presented in black size 40 Arial font 10^∘^ of visual angle to the left or right of the center of the screen in the overt and covert attentional shift conditions, or at the center of the screen in the central fixation condition. Chevrons were presented at a rate of one every 250 ms. Participants were required to press space bar to detect sideways chevrons in all conditions. In the overt and covert attentional shift conditions, the side of presentation flipped immediately after a sideways chevron was shown. In the overt attentional interference task type condition, participants were required to shift their gaze to fixate the chevrons. In the covert attentional interference task type condition, participants were required to orient attention to the contralateral channel and detect the sideways facing chevrons while maintaining fixation on the central fixation cross. In the central fixation condition, participants were required to detect sideways facing chevrons presented in the center of the screen.

Participants’ gaze was monitored using a tower-mounted EyeLink 1000 eye tracker (SR Research). Stimuli were presented on a 20-in. CRT screen with a refresh rate of 85 Hz. Participants sat 60 cm from the computer screen, with the center of screen at eye level.

#### Procedure

Participants were instructed to maintain fixation on the center of the screen throughout presentation of the array. They were specifically informed that they should remember information about the colored dots on screen. They were also instructed to either shift their gaze (overt attentional task type condition) or to maintain central fixation and detect chevrons appearing either to the left and right of the center of the screen or at the center of the screen throughout the delay period.

**Fig. 1:**
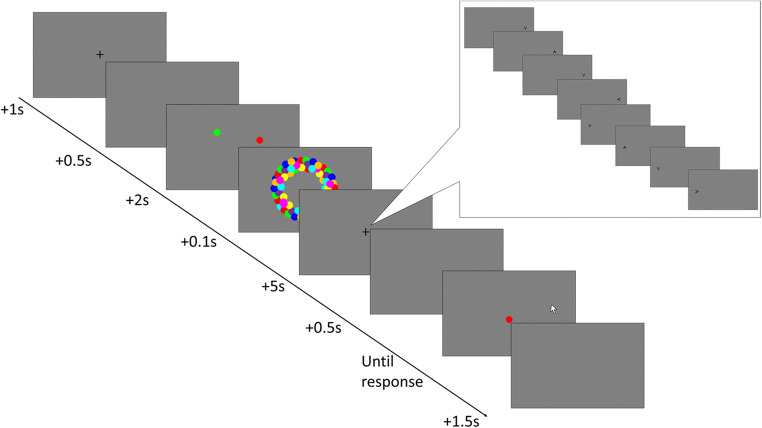
An example trial for each condition in Experiment 1. Participants were shown an array of between one and four colored dots. During the delay period, participants were required to complete a detection task at either central fixation or in peripheral vision by making saccades or covertly shifting attention. The detection task for saccadic and attentional interference conditions is shown in the bubble. After this delay, they were shown one of the colored dots in the center of the screen and asked to click on screen where that dot first appeared

Trials began with the presentation of a fixation cross at the center of the screen for 1,000 ms. The fixation cross was followed by a blank screen for 500 ms. The fixation cross and the stimulus array, comprising one, two, or four colored dots, were presented for 2,000 ms. After presentation of the array, the visual mask was presented for 100 ms. Participants then performed the detection task for 5,000 ms. Following this delay period, one of the stimuli from the array was randomly chosen and presented in the center of the screen. Participants were required to move the mouse to click the location on screen where it first appeared. After participants submitted their response, their response was shown on-screen for 500 ms. No other feedback was given in experimental trials. There was no time limit for responding, but participants were asked to respond as quickly and as accurately as possible. A 1,500-ms blank screen followed the response screen, before the beginning of the next trial. After each block of 15 trials, participants were permitted to take a self-paced break. An example trial is shown in Fig. 1.

## Experiment 2

Experiment 2 attempted to examine whether there were analogous oculomotor interference effects in working memory for non-spatial features as in spatial working memory. We proposed the following hypotheses, based on the proposal that the oculomotor system is selectively involved in maintenance of spatial information in VSWM (Pearson et al., 2014):

1. There will be a small effect of interference task type on misbinding due to participants performing a dual-task, with the possibility that misbinding will be highest for the overt attentional shift condition as activation of the oculomotor system may disrupt the memory of the spatial location, which has been proposed to aid maintenance of non-spatial features in VSWM (Schneegans & Bays, 2017). This interference of the spatial representations due to oculomotor activity may in turn disrupt memory for the color-location binding. Furthermore, we predict that there may be a small effect of interference task type on guessing, where guessing will increase in the attentional shift conditions due to performing a dual-task. However, we predict that there will be no significant effect of interference task type on imprecision, and that the effect of interference task type on imprecision will be smaller in this experiment compared to the effect of interference task type on imprecision in Experiment 1, based on the proposal that the oculomotor system is selectively involved in maintenance of spatial locations in VSWM.
2. There will be a significant main effect of set size, such that imprecision and misbinding will increase with increases in the number of to-be-remembered items (Bays et al., 2009).

We also explored the interaction between set size and interference task type, but made no specific hypotheses regarding this effect.

### Method

#### Participants

Based on the power analysis carried out to detect the interaction between task and interference type (Experiment 1, Hypothesis 3), we required a sample of 12 participants for this experiment. Data collection was terminated when 12 usable datasets were obtained. Undergraduate participants received participant pool credit for their time. We received ethics approval from Department of Psychology Research Ethics Committee (reference: PSYCH-2019-10-28T15:23:58-lckd86).

#### Design

The design was the same as Experiment 1.

#### Stimuli

Stimuli were the same as Experiment 1, with the exception that, at recall, a color wheel, with depth 2^∘^ of visual angle, was presented 11^∘^–13^∘^ of visual angle around central fixation.

#### Procedure

**Fig. 2:**
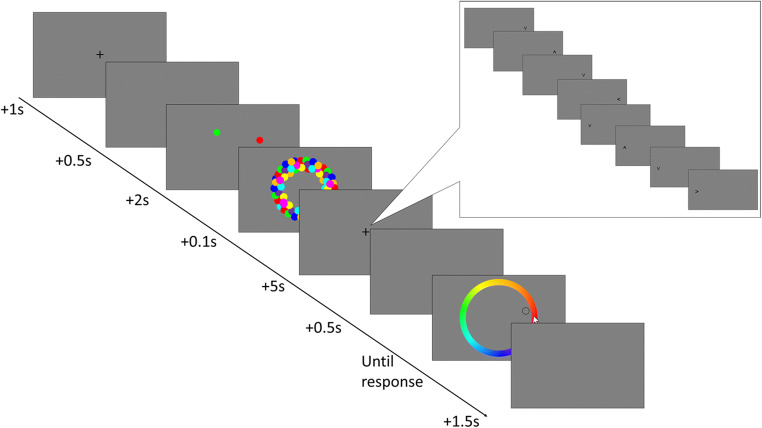
An example trial for each condition in Experiment 2. This is the same as Experiment 1 with the exception that participants were shown one of the dots in their original location and were asked to click on a color wheel the original color of the probe

The procedure followed Experiment 1, with the exception that, at recall, participants were shown the outline of one of the dots in their original location and were required to click the color on the color wheel that matched the color originally presented at the probed location (see Fig. 2).

## Statistical analysis

For each experiment, we excluded any trials in which eye position was outside of an interest period with radius 2° of visual angle around central fixation during the encoding period in all conditions, and during the delay period for the maintained fixation and covert attention interference task type conditions. Based on previous studies, around 20% of trials in our studies are excluded due to excessive eye movements. Additionally, participants were required to correctly detect more than 50% of the presented sideways-facing chevrons during the interference task. Therefore, a usable dataset was defined as one in which there were at least 120 trials per set size in each interference task after controlling for performance in the delay-period task and eye movements. This minimum number of trials is greater than the minimum number of trials required for the mixture modelling to be considered reliable (Bays et al., 2009).

We applied Bays et al.’s (2009) mixture modelling to the data for each experiment using the Matlab functions provided by Bays et al. (2009) and Grogan et al. (2020; Suchow et al., 2013). This modelling fits a mixture of normal and uniform distributions to the response data. The normal distributions are centered on the probed stimulus, representing the probability of reporting the target location, and the uniform distribution represents guessing, where all possible responses are equally likely. Additional normal distributions, centered on the non-probed stimuli, were also fit to the data to retrieve a measure of misbinding. The standard deviation of the normal distributions gives a measure of imprecision: the wider the distribution, the less precise (more variable) the response. The heights of each of the distributions represent response likelihood, between zero and one, for reporting the target feature, a non-target feature, and guessing. In Experiment 1, the distributions were centered on the spatial location of the probed stimulus, therefore a bivariate Gaussian distribution was fit to the data (Grogan et al., 2020). In Experiment 2, the distributions were centered on the angle on the color wheel of the probed stimulus, therefore a Von Mises distribution was fit to the data (Bays et al., 2009).

For each experiment, we analyzed the imprecision, and the probabilities of reporting the target, misbinding and guessing using separate 3 x 3 repeated-measures ANOVA with factors of set size (three levels: one, two, four items) and interference task type (three levels: saccadic interference, covert attention, maintained fixation). If the assumption of sphericity was violated, Greenhouse-Geisser correction was applied. These analyses were carried out in R (R Core Team, 2019) using the rstatix package (Kassambara, 2019).

For a significant main effect of set size, we carried out pairwise comparisons, comparing set size one to set size two, and set size two to set size four. For a significant main effect of interference task type, we compared all interference tasks. To examine a significant interaction between set size and interference task type, we examined the differences between interference tasks at each set size. Holm-Bonferroni correction was applied to control for multiple comparisons.

We also directly compared the effects of interference task types between Experiments 1 and 2 for imprecision to examine the hypothesis that the oculomotor system is specifically involved in rehearsal of spatial information in VSWM. The effects of interference type were collapsed across set sizes and analyzed using a 2 x 3 mixed ANOVA with between-subjects factor of task (two levels: spatial and color) and within-subjects factor of interference task type (three levels: saccadic interference, covert attention, maintained fixation). If a significant interaction was observed, we examined the differences between each interference task condition for each task.

## Results

### Experiment 1

Fifteen participants (*M* = 19.33 years, *SD* = 1.18, 12 females, three males, 13 right-handed, one left-handed, one ambidextrous, all confirmed normal or corrected-to-normal vision) volunteered and completed this experiment.^1^ After acceptance, participants were unable to meet our original inclusion criteria. Due to differences in reaction times affecting the precision of when the press was detected, performance on the detection task was calculated by the total number of times the space bar was pressed, divided by the number of times a sideways-facing chevron was presented. Participants were unaware of this change in calculation and were instructed to perform the detection task as quickly and as accurately as possible. Additionally, participants were unable to meet our original target of at least 120 valid trials per set size in each condition, so this was relaxed to at least 50 valid trials per set size in each condition. This resulted in the exclusion of three datasets from analysis. Of the remaining 12 datasets, 30.58% of trials were excluded.

#### Detection-task analysis

We examined whether performance in the detection task varied according to the delay-period interference task type. Overall, the average performance on the detection task was 0.87 (*SD* = 0.07). Performance on the detection task did not differ across interference task types; *F*(2, 22) = 1.76, *p* = 0.195, ${\eta}_p^2$ = 0.14.

#### Mixture modelling

**Fig. 3:**
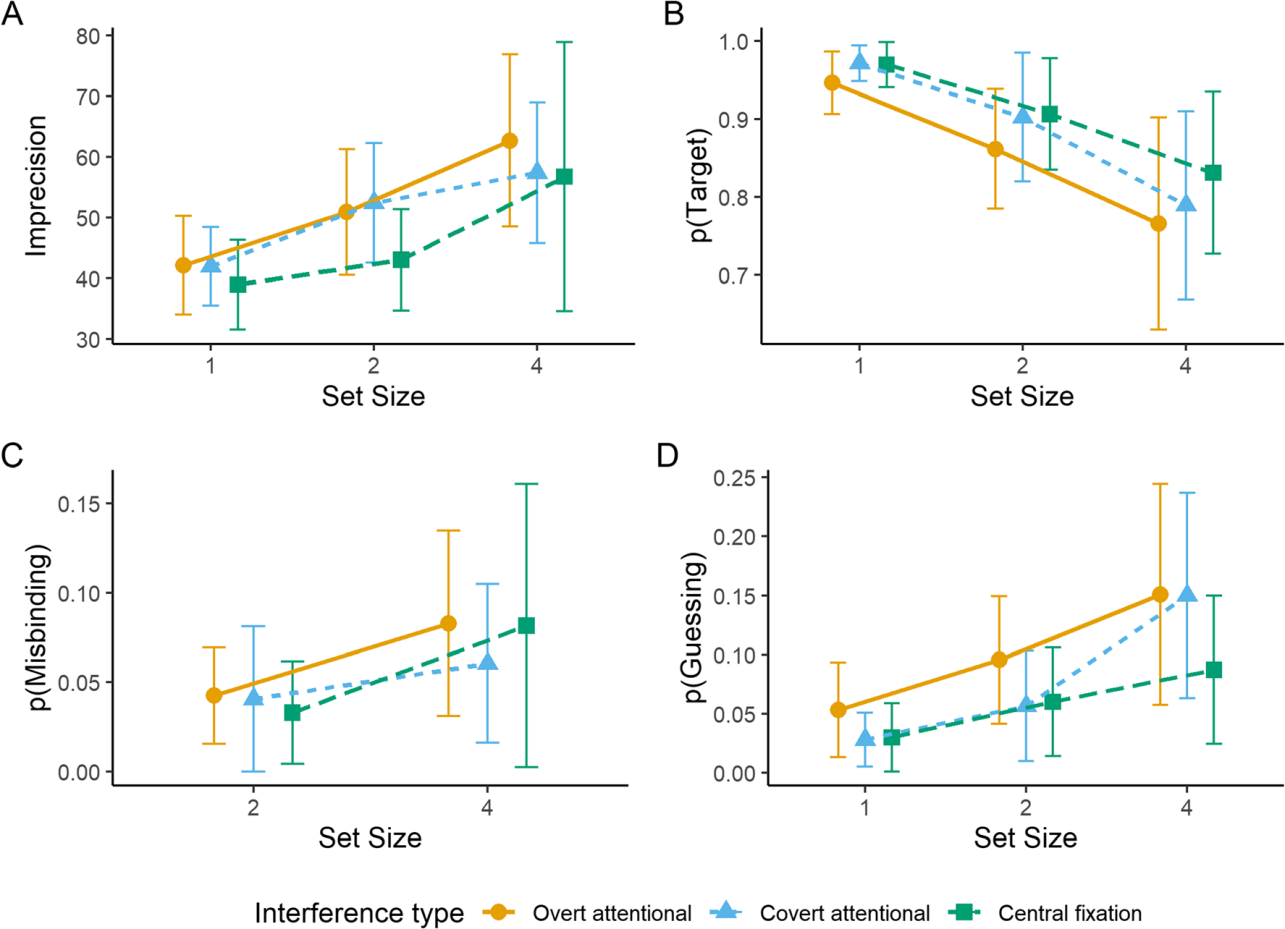
Mean imprecision (**A**), mean probability of reporting the target location (**B**), mean probability of reporting a non-target (misbinding; **C**), and mean probability of guessing (**D**) as a function of set size in each interference condition. Error bars represent 95% confidence intervals

For imprecision (Fig. 3A), a 3 x 3 repeated-measures ANOVA revealed a significant main effect of set size; *F*(1.3, 14.25) = 15.27, *p* < 0.001, ${\eta}_p^2$ = 0.58. Neither the main effect of interference task type [*F*(2, 22) = 1.72, *p* = 0.202, ${\eta}_p^2$ = 0.14] nor the interaction between interference task type and set size [*F*(1.52, 16.7) = 0.79, *p* = 0.436, ${\eta}_p^2$ = 0.07] were significant. Holm-Bonferroni corrected pairwise comparisons between set sizes revealed that the difference between set size one (*M* = 41.01, *SD* = 12.08) and set size two (*M* = 48.79, *SD* = 16.24) was significant; *t*(35) = -4.75, *p* < 0.001. The difference between set size two and set size four (*M* = 58.94, *SD* = 27.2) was also significant; *t*(35) = -3.52, *p* = 0.001.

For the probability of reporting the target location (Fig. 3B), repeated-measures ANOVA revealed significant main effects of set size [*F*(1.12, 12.3) = 14.22, *p* = 0.002, ${\eta}_p^2$ = 0.56] and of interference task type [*F*(2, 22) = 4.93, *p* = 0.017, ${\eta}_p^2$ = 0.31]. The interaction between interference task type and set size was not significant; *F*(4, 44) = 0.91, *p* = 0.469, ${\eta}_p^2$ = 0.08. For the main effect of set size, Holm-Bonferroni corrected pairwise comparisons between set sizes revealed that the difference between set size one (*M* = 0.96, *SD* = 0.05) and set size two (*M* = 0.89, *SD* = 0.13) was significant; *t*(35) = 4.64, *p* < 0.001. The difference between set size two and set size four (*M* = 0.8, *SD* = 0.2) was also significant; *t*(35) = 5.82, *p* < 0.001. For the main effect of interference task type, the probability of reporting the target location was significantly lower in the overt attentional interference task (*M* = 0.86, *SD* = 0.17) compared to covert attentional interference task (*M* = 0.89, *SD* = 0.16; *t*(35) = -2.76, *p* = 0.018), and compared to when central fixation was maintained (*M* = 0.9, *SD* = 0.14; *t*(35) = -3.15, *p* = 0.010). The difference between covert attentional interference task and central fixation was not significant; *t*(35) = -1.18, *p* = 0.245.

For the probability of misbinding (Fig. 3C), a repeated-measures ANOVA revealed a significant main effect of set size; *F*(1, 11) = 4.95, *p* = 0.048, ${\eta}_p^2$ = 0.31. That is, the difference between set size two (*M* = 0.04, *SD* = 0.06) and set size four (*M* = 0.07, *SD* = 0.1) was significant. Neither the main effect of interference task type [*F*(2, 22) = 0.59, *p* = 0.565, ${\eta}_p^2$ = 0.05] nor the interaction between interference task type and set size [*F*(2, 22) = 1.26, *p* = 0.304, ${\eta}_p^2$ = 0.1] were significant.

For the probability of guessing (Fig. 3D), a repeated-measures ANOVA revealed significant main effects of set size [*F*(1.22, 13.38) = 15.32, *p* = 0.001, ${\eta}_p^2$ = 0.58] and interference task type [*F*(2, 22) = 4.14, *p* = 0.030, ${\eta}_p^2$ = 0.27]. The interaction between interference task type and set size was not significant; *F*(1.53, 16.85) = 1.64, *p* = 0.224, ${\eta}_p^2$ = 0.13. For the main effect of set size, Holm-Bonferroni corrected pairwise comparisons between set sizes revealed that the difference between set size one (*M* = 0.04, *SD* = 0.05) and set size two (*M* = 0.07, *SD* = 0.08) was significant; *t*(35) = -3.68, *p* < 0.001. The difference between set size two and set size four (*M* = 0.13, *SD* = 0.14) was also significant; *t*(35) = -4.15, *p* < 0.001. For the main effect of interference type, the probability of guessing was significantly higher in the overt attentional interference task condition (*M* = 0.1, *SD* = 0.12) compared to the covert attentional interference (*M* = 0.08, *SD* = 0.11; *t*(35) = 2.57, *p* = 0.034), and compared to when central fixation was maintained (*M* = 0.06, *SD* = 0.08; *t*(35) = 2.67, *p* = 0.034). The difference between the covert attentional interference task and central fixation was not significant; *t*(35) = 1.31, *p* = 0.199.

#### Directional-specificity of localisation error (exploratory analysis)

**Fig. 4:**
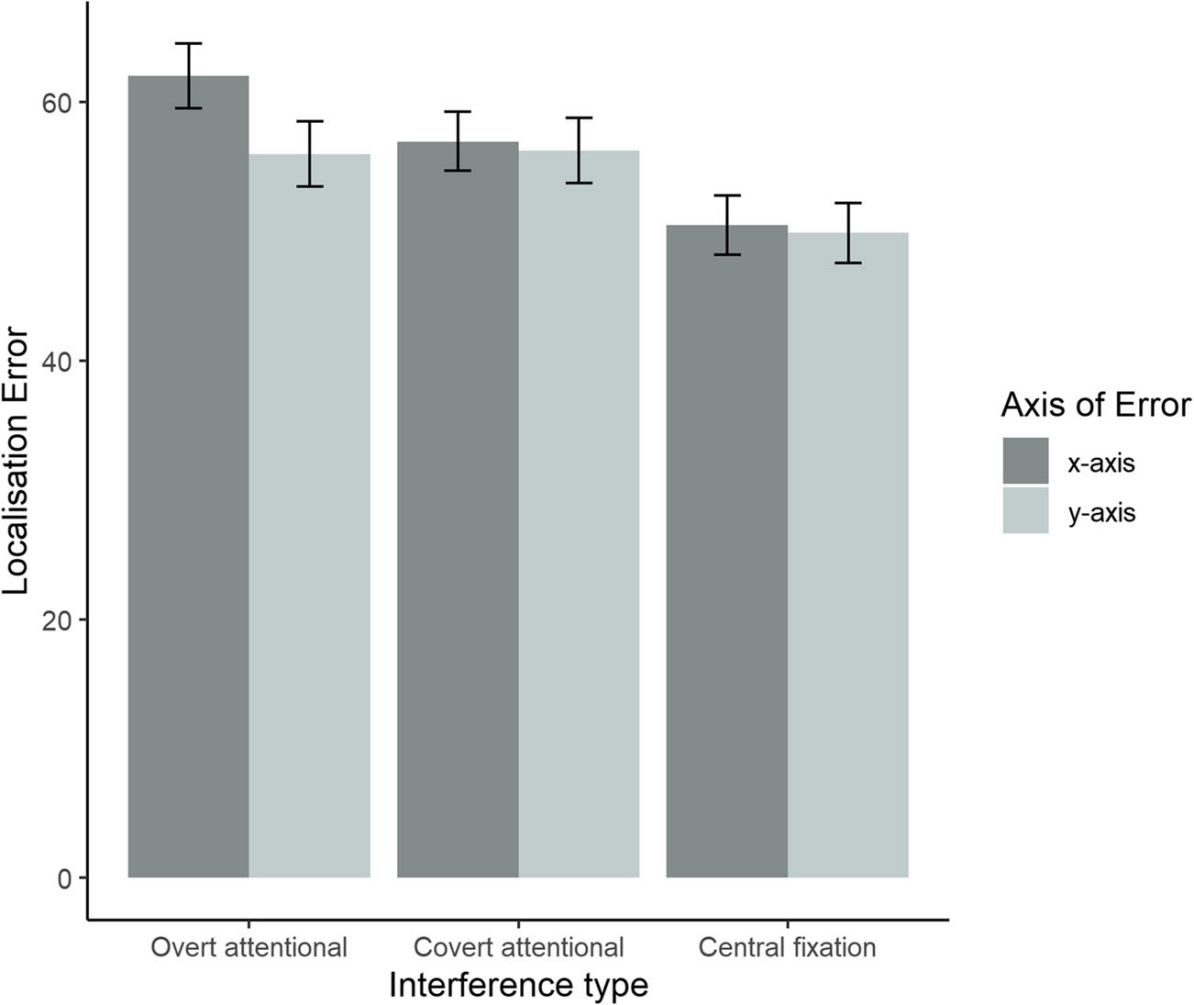
Mean localization error for each axis of response in each interference condition. Error bars represent 95% confidence intervals

We also examined whether there was a directionally specific effect of interference task type on localization error. Because responses were two dimensional, along the x- and y-axes, we analyzed the error along these axes separately using a factor called axis of error. We carried out a 3 x 3 x 2 repeated-measures analysis of variance with factors of interference task type (3 levels: overt attentional, covert attentional interference, central fixation), set size (3 levels: one, two, and four items), and axis of error (2 levels: x-axis, y-axis). Significant effects of set size [*F*(1.1, 12.07) = 15.86, *p* = 0.001, ${\eta}_p^2$ = 0.59] and interference task type [*F*(2, 22) = 5.48, *p* = 0.012, ${\eta}_p^2$ = 0.33] were observed. A significant two-way interaction between axis of error and interference task type was also observed; *F*(2, 22) = 5.19, *p* = 0.014, ${\eta}_p^2$ = 0.32. Examination of this interaction revealed that localisation error along the x-axis (*M* = 62.03, *SD* = 94.37) was significantly greater than localisation error along the y-axis (*M* = 55.98, *SD* = 93.85) in the overt attentional shift condition; *t*(35) = 4.26, *p* < 0.001. The difference in localisation error along the x- and y-axes were not significant in the covert attention [*t*(35) = 0.38, *p* = 0.703] and central fixation conditions [*t*(35) = 0.48, *p* = 0.634]. No other effects were significant; *p* ≥ 0.054. These results are illustrated in Fig. 4.

#### Discussion

This experiment aimed to examine how overt and covert attentional interference tasks affected the representation of spatial information in VSWM. We found that the probability of guessing was highest when the oculomotor system was activated compared to when attention was covertly shifted and when central fixation was maintained. There was no significant difference in the probability of guessing between the covert attentional shift condition and maintaining central fixation. This result offered some support for Hypothesis 1. However, there was no significant effect of interference task type on imprecision or misbinding, which is inconsistent with the predictions outlined in Hypothesis 1. We also observed that imprecision and misbinding increased with increases in set size, consistent with the predictions of Hypothesis 2. The probability of guessing also increased with increases in set size, which was not predicted in any of our hypotheses. These findings of an effect of set size on imprecision, misbinding, and guessing are consistent with previous work supporting that VSWM is a flexible and dynamic resource (Bays et al., 2009). No significant interaction between interference task type and set size was found, indicating that the effects of interference task types do not differ as the number of memoranda differ.

The observation that guessing was highest following saccadic shifts compared to covert attentional shifts and maintaining central fixation is consistent with previous work (Peterson et al., 2019; Schut et al., 2017). However, the lack of an effect of interference type on imprecision is surprising, and not consistent with previous findings, which show a decrease in precision in VSWM following saccadic shifts (Peterson et al., 2019; Schut et al., 2017). The results of this experiment indicate that performing multiple task-irrelevant saccades does not impact the quality of the representation of spatial locations in VSWM; when participants are reporting an item that is represented in VSWM, they are relatively accurate. However, saccadic interference affects the probability of memory items being represented in VSWM. Specifically, it appears that producing a series of delay-period saccades results in the removal of information from VSWM.

We also explored whether there was a directionally specific effect of interference task type on response error. In line with previous work, response error was greatest along the axis of the saccade (Peterson et al., 2019). This result seems consistent with their proposal that spatial memory representations are updated when a saccade is executed, but that the updating process is noisy, leading to a loss of fidelity. Unlike Peterson et al. (2019), we observed no effect of saccades on precision per se. It is possible that a subtle, directional effect of saccadic interference on imprecision was hidden because the effects were collapsed across x- and y-axes to obtain a single measure of imprecision in spatial working memory. Nonetheless, our finding of a directionally specific increase in localisation error, which can be used as a proxy for precision, indicates a directionally specific loss in precision that is specific to activation of the oculomotor system. It is also possible that this difference reflects differences in the reference frame required for our task. Specifically, our task required localisation of the memoranda, which may have engaged a more egocentric frame of reference, whereas that of Peterson et al. (2019) relied primarily on an allocentric frame to recall the relative positions of the stimuli.

### Experiment 2

Seventeen participants (*M* = 20.35 years, *SD* = 2.42, 13 females, four males, 17 right-handed, all confirmed normal or corrected-to-normal vision) volunteered and completed this experiment.^2^ Five participants were excluded from analysis because they did not meet the revised inclusion criteria, as outlined in Experiment 1. Of the remaining 12 datasets, 29.54% of trials were excluded.

#### Detection-task analysis

We examined whether performance in the detection task varied according to the delay-period interference task type. Overall, average performance on the detection task was 0.86 (*SD* = 0.07) and this did not differ across interference task types; *F*(1.15, 12.63) = 2.45, *p* = 0.140, ${\eta}_p^2$ = 0.18.

#### Mixture modelling

**Fig. 5:**
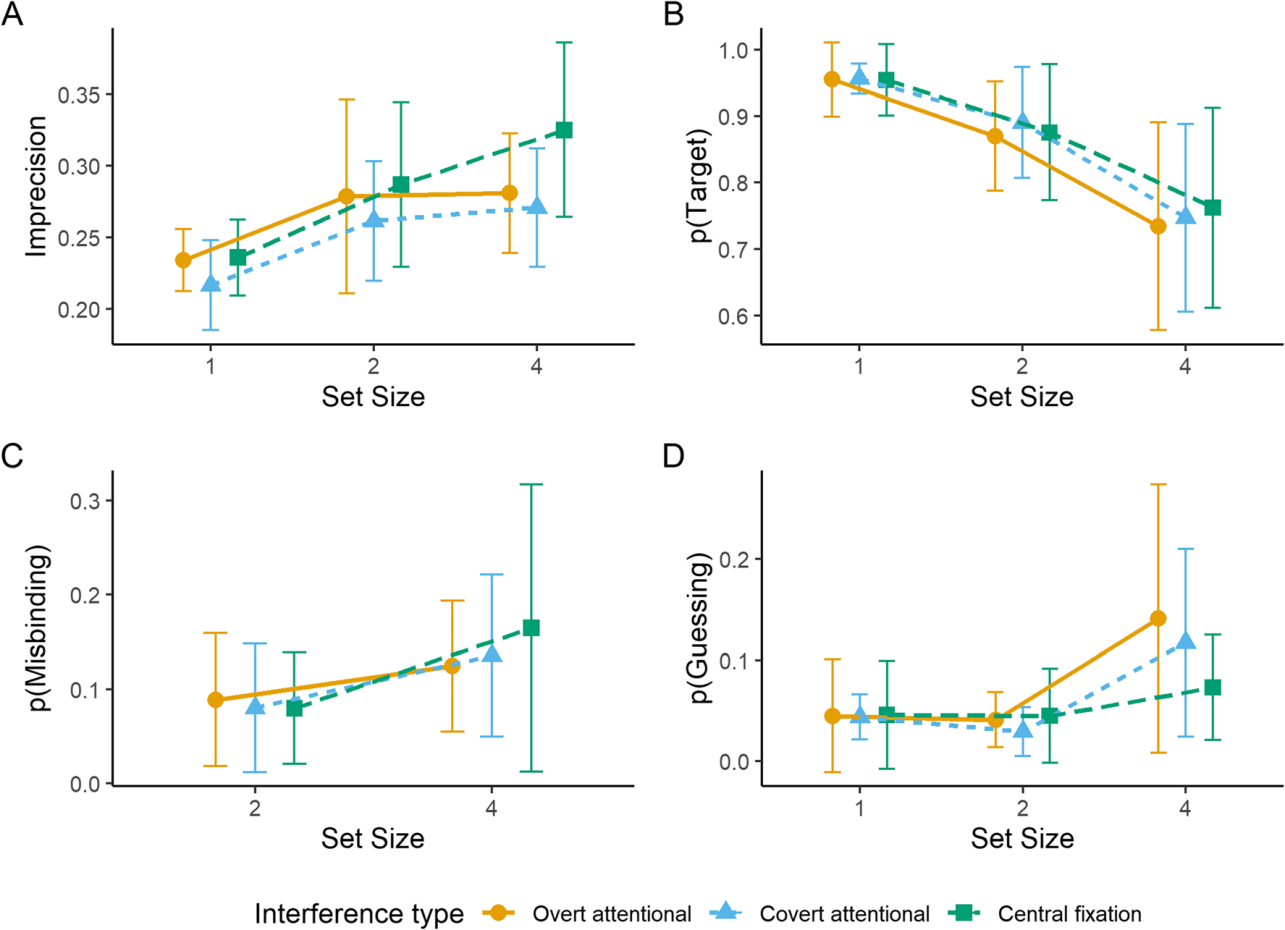
Mean imprecision (**A**), mean probability of reporting the target location (**B**), mean probability of reporting a non-target (misbinding; **C**), and mean probability of guessing (**D**) as a function of set size in each interference condition. Error bars represent 95% confidence intervals

Imprecision (Fig. 5A) for color data was calculated by transforming the concentration factor (*κ*) of the circular normal distribution to the circular standard deviation. A repeated-measures ANOVA revealed a significant main effect of set size; *F*(2, 22) = 8, *p* = 0.002, ${\eta}_p^2$ = 0.42. Neither the main effect of interference task type [*F*(1.31, 14.4) = 3.02, *p* = 0.096, ${\eta}_p^2$ = 0.22] nor the interaction between interference task type and set size [*F*(2, 21.96) = 0.57, *p* = 0.572, ${\eta}_p^2$ = 0.05] were significant. For the main effect of set size, Holm-Bonferroni corrected pairwise comparisons between set sizes revealed that the difference between set size one (*M* = 0.23, *SD* = 0.04) and set size two (*M* = 0.28, *SD* = 0.09) was significant; *t*(35) = -3.59, *p* = 0.002. The difference between set size two and set size four (*M* = 0.29, *SD* = 0.08) was not significant; *t*(35) = -1.04, *p* = 0.307.

For the probability of reporting the target location (Fig. 5B), a repeated-measures ANOVA revealed a significant main effect of set size; *F*(1.12, 12.28) = 15.08, *p* = 0.002, ${\eta}_p^2$ = 0.58. Neither the main effect of interference task type [*F*(2, 22) = 0.51, *p* = 0.608, ${\eta}_p^2$ = 0.04] nor the interaction between interference task type and set size [*F*(2.13, 23.44) = 0.52, *p* = 0.615, ${\eta}_p^2$ = 0.04] were significant. For the main effect of set size, Holm-Bonferroni corrected pairwise comparisons between set sizes revealed that the difference between set size one (*M* = 0.96, *SD* = 0.08) and set size two (*M* = 0.88, *SD* = 0.15) was significant; *t*(35) = 5.05, *p* < 0.001. The difference between set size two and set size four (*M* = 0.75, *SD* = 0.24) was also significant; *t*(35) = 6.29, *p* < 0.001.

For the probability of misbinding (Fig. 5C), a repeated-measures ANOVA revealed a significant main effect of set size; *F*(1, 11) = 12.22, *p* = 0.005, ${\eta}_p^2$ = 0.53. That is, the difference between set size two (*M* = 0.08, *SD* = 0.11) and set size four (*M* = 0.14, *SD* = 0.18) was significant. Neither the main effect of interference task type [*F*(1.2, 13.16) = 0.3, *p* = 0.631, ${\eta}_p^2$ = 0.03] nor the interaction between interference task type and set size [*F*(1.1, 12.13) = 0.46, *p* = 0.531, ${\eta}_p^2$ = 0.04] were significant.

For the probability of guessing (Fig. 5D), a repeated-measures ANOVA revealed a significant main effect of set size; *F*(1.15, 12.63) = 7.17, *p* = 0.017, ${\eta}_p^2$ = 0.39 . Neither the main effect of interference task type [*F*(2, 22) = 0.89, *p* = 0.425, ${\eta}_p^2$ = 0.07] nor the interaction between set size and interference task type [*F*(1.24, 13.69) = 0.82, *p* = 0.408, ${\eta}_p^2$ = 0.07] were significant. For the main effect of set size, Holm-Bonferroni corrected pairwise comparisons between set sizes revealed that the difference between set size two (*M* = 0.04, *SD* = 0.06) and set size four (*M* = 0.11, *SD* = 0.16) was significant; *t*(35) = -2.91, *p* = 0.012. The difference between set size one (*M* = 0.04, *SD* = 0.08) and set size two was not significant; *t*(35) = 0.76, *p* = 0.454.

A summary of the key effects in Experiments 1 and 2 is presented in Table 1.

**Table 1:** Summary table of significance levels of each variable in each experiment

Experiment	Dependent variable	Set size	Interference task type	Set size x interference task type interaction
Experiment 1: Space	Imprecision	< 0.001***	0.202	0.436
	pTarget	0.002**	0.017*	0.469
	pMisbind	0.048*	0.565	0.304
	pGuess	0.001***	0.03*	0.224
Experiment 2: Colour	Imprecision	0.002**	0.096	0.572
	pTarget	0.002**	0.608	0.615
	pMisbind	0.005**	0.631	0.531
	pGuess	0.017*	0.425	0.408

* indicates *p* < 0.05, ** indicates* p* < 0.01, *** indicates* p* < 0.001

#### Comparison between Experiment 1 and Experiment 2

We then examined whether the effects of the interference task types significantly differed from each other across experiments (i.e., whether there was a significant experiment x interference task type interaction). The imprecision data from Experiment 1 were transformed such that the area of the response space was equal to 360 to enable better comparison with the data from Experiment 2. Mixed-factor ANOVA revealed that the interaction between experiment and interference task type was not significant; *F*(2, 44) = 2.67, *p* = 0.080, ${\eta}_p^2$ = 0.11.

#### Discussion

This experiment aimed to examine whether a performing delay-period saccades impacted memory for color in a similar way to the effects on memory for spatial locations. We found no significant effects of interference task type on any outcome variable, consistent with the predictions of Hypothesis 1. We observed reliable increases in imprecision and misbinding increased with increases in set size, consistent with the predictions outlined in Hypothesis 2. We also observed a significant effect of set size on guessing. These findings are consistent with previous work examining memory for color (Bays et al., 2009). Additionally, there was no interaction between interference task type and experiment on imprecision (Experiment 1, Hypothesis 3), indicating that the overt attentional interference task did not have a greater effect on the precision with which spatial locations are maintained in VSWM compared to the precision with which non-spatial features are maintained. This lack of an interaction between interference task type and experiment is perhaps unsurprising given the non-significant effect of interference task type on imprecision in Experiment 1.

The lack of an effect of interference task type on misbinding was not consistent with the predictions in Hypothesis 1. The original rationale for this hypothesis arose from the fact that participants were required to compare the probed location with the stored location to retrieve the correct color. This aspect of the design means any changes in precision of the representation of the probed location could lead to increased misbinding errors in the color task (Hypothesis 1). However, performing delay-period saccades did not result in an increase in imprecision in memory for spatial location in Experiment 1, and as a consequence there was no effect on the misbinding in the color task.

To summarize the key findings from Experiment 2, no effect of interference task type was observed when examining any outcome variable. This is in contrast to Experiment 1, where performing delay-period saccades resulted in an increase in guessing. This result confirms that saccadic disruption is not observed when probing non-spatial features in VSWM (Ball et al., 2013; Pearson et al., 2014; Pearson & Sahraie, 2003; Postle et al., 2006).

## General discussion

Previous work has shown that saccadic eye movements during the maintenance of spatial locations leads to an impairment of VSWM (Pearson & Sahraie, 2003; Peterson et al., 2019). This impairment is specific to spatial features and is greater than and independent of the interference caused by covert shifts of attention (Lawrence et al., 2004; Pearson et al., 2014). The current study investigated the mechanisms underlying this selective interference effect by examining how delay-period activation of the oculomotor system affected representations of spatial and non-spatial (visual) features in VSWM. Critically, saccadic eye movements resulted in an increase in the probability of guessing when spatial locations were probed (Experiment 1) but not when color memory was examined (Experiment 2). Covert shifts in attention had no effect on memory representations in either experiment. Furthermore, saccadic eye movements did not disrupt the precision with which spatial and visual features are maintained. However, in both experiments imprecision, misbinding and guessing increased with increases in set size.

These findings are broadly consistent with previous work showing that delay-period activation of the oculomotor system interferes with spatial but not visual working memory (Ball et al., 2013; Pearson et al., 2014). Our data cannot be explained by the removal of attention because our delay-period detection task required either a covert or overt shift of attention. If rehearsal in VSWM takes place via shifts in attention (Awh & Jonides, 2001), performance on the covert and overt attentional interference conditions would have been predicted to be relatively equal. In contrast, we observed that guessing responses were significantly higher in the overt than the covert attention interference task condition. Additionally, guessing in the covert attentional interference task condition was not significantly different from maintaining central fixation. This pattern of data indicates that it is activation of the oculomotor system and not shifts of attention alone that drives an increase in guessing. The increase in guessing in spatial memory found in Experiment 1, but no increase in misbinding in Experiment 2, following saccadic movements is also consistent with Pearson and Sahraie (2003). When comparing the ratio of temporal errors with spatial errors across conditions, they showed significantly more spatial errors than temporal errors in conditions involving eye movements. Additionally, conditions involving eye movements produced significantly more spatial errors than shifts of covert attention alone (Pearson & Sahraie, 2003). Peterson et al. (2019) have also shown an increase in guessing following performance of delay-period saccades. Our findings and those of previous studies (Pearson & Sahraie, 2003; Peterson et al., 2019), therefore suggest that the mechanism by which delay-period saccades interfere with VSWM is by increasing guessing within spatial working memory.

Our exploratory analysis in Experiment 1, examining the directional-specificity of interference task types on response error indicated that response error was greater along the axis of the saccade compared to the axis orthogonal to the saccade. This effect was not found when the oculomotor system was not activated, indicating that the effect is specific to the activation of the oculomotor system. This result lends support for the idea that performing saccades leads to noisy updating of the spatial maps that represent memory items (Peterson et al., 2019), with the caveat that we did not observe significant effects of saccadic interference on precision when collapsed across x- and y-axes.

Our data showed an increase in guessing with no corresponding increase in imprecision in Experiment 1. One way to interpret the pattern of our data might be in terms of a slot model of VSWM (Zhang & Luck, 2008). According to a slot model, items are stored in a finite number of independent slots. Several recent studies have shown that the goal of an eye movement is automatically encoded into VSWM, irrespective of its task relevance (Schut et al., 2017; Tas et al., 2016). In the current task, memory items were encoded with the eyes fixated, then participants made saccades to the task-irrelevant locations. If these saccades led to the automatic encoding of the saccade targets, they would fill two of the available slots. This would result in VSWM representations being removed from two slots, which would manifest as an increase in guessing. Because the slots are independent, this removal would not affect the precision with which items in other slots are retained, consistent with the pattern of increased guessing in the absence of reduced precision.

However, there are reasons to be cautious in accepting this interpretation. Specifically, the slot model assumes that items are held as independent bound representations and that spatial and non-spatial features are held in the same representation. This model therefore predicts that there should be no misbinding of features. In contrast to this prediction, we found a reliable increase in misbinding with increases in set size, consistent with previous research in spatial (Schneegans & Bays, 2016) and visual working memory (Ma et al., 2014). Furthermore, contrary to the bound-representation assumption, we found that the overt attentional interference task interfered with spatial but not visual working memory, indicating that these features may be maintained somewhat independently. Indeed, these effects are more consistent with the resource model of VSWM (Bays et al., 2009), which proposes that precision is dependent on the proportion of resources dedicated to each item and that visual and spatial features are held independently (Bays et al., 2011). We also observed a set size effect on precision for both spatial and visual memory, which is more consistent with resource than slot models.

The resource model appears to offer the best explanation for the majority of the results. However, it remains difficult to explain why guessing increased, without a corresponding increase in imprecision, in the overt attentional interference task condition in spatial working memory with this model. One speculative proposal is that VSWM items were encoded with a high degree of precision. When the saccade targets were encoded into VSWM during maintenance, resources were removed from VSWM items. However, this did not significantly affect the precision with which VSWM items were retained because the initial representation was encoded with such a high degree of precision. It may be that the effect of performing saccades on precision was limited due to the small range of set sizes used in this study. It is possible that as more items are to be retained and less resource is directed to each item, the effects of performing saccades would be greater on precision.

An alternative explanation might be that delay-period oculomotor activation disrupts the ability to discriminate signals from noise in spatial working memory. This would be problematic, because the resource model assumes that VSWM representations are noisy but have a high signal-to-noise ratio, resulting in responses falling around the probed item. Consistent with this, patients with Parkinson’s disease show increased guessing responses with little difference in precision in orientation memory (Zokaei et al., 2014), which was attributed to reduced signal to noise ratio caused by dopamine depletion in Parkinson’s disease. As has already been noted, saccadic eye-movements require the updating of the internal spatial maps that maintain representations of spatial locations. This updating is likely to be imperfect, which may plausibly result in a reduction of signal-to-noise in the maps. Reduced signal-to-noise would result in an inability to retrieve the correct information at recall, leading to increased guessing. However, because changing signal-to-noise does not affect the allocation of resource to the memoranda, precision of correctly recalled locations is unaffected, consistent with the data observed in Experiment 1.

To summarize, we have demonstrated that interference in VSWM due to delay-period saccades are specific to spatial, but not visual, working memory, and that the disruptive effect arises from an increase in guessing rather than a reduction in the precision with which spatial locations are retained in VSWM. On first inspection the finding that disruption following saccades leads to increased guessing but not decreased precision appears to argue for a slot-based model of VSWM. However, we also observed increased misbinding and imprecision as set size increased and a dissociation in saccadic interference effects between spatial and color working memory, which is more consistent with resource models. To reconcile the effect of guessing with the resource model, it was proposed that delay-period saccadic movements led to reduced signal-to-noise ratio in spatial working memory, thus increasing the probability that responses fell far from the location of the probed item.

## Footnotes

1 An additional six participants volunteered but withdrew during or after their first testing session. Their data were discarded upon their withdrawal from the study.

2 An additional five participants volunteered but withdrew during or after their first testing session. Their data were discarded upon their withdrawal from the study.
